# Artificial Intelligence in Surgical Documentation: A Critical Review of the Role of Large Language Models

**DOI:** 10.1007/s10439-023-03282-2

**Published:** 2023-06-20

**Authors:** Aaron Lawson McLean

**Affiliations:** Department of Neurosurgery, Jena University Hospital – Friedrich Schiller University Jena, Am Klinikum 1, 07747 Jena, Germany

**Keywords:** Artificial intelligence, Surgical documentation, Operative notes, GPT-4, Data protection, Healthcare applications

## Abstract

This article provides a critical analysis of the application of the advanced language model, GPT-4, in generating surgical operative notes, with a focus on its use in ophthalmology as presented by Waisberg et al. The discussion underscores the inherent complexity and specificity of operative notes, the issue of accountability, and the potential data protection issues associated with the use of AI in healthcare. The letter emphasizes the need for a more comprehensive understanding of the complexities involved in applying AI in healthcare and calls for a more nuanced and responsible approach to the integration of AI in surgical documentation.

Dear Editor,

I am writing to express my concerns regarding the article "GPT-4 and Ophthalmology Operative Notes" by Waisberg et al. [1]. The authors propose the use of GPT-4, an advanced large language model developed by OpenAI, to generate ophthalmic operative notes.

While the potential of AI in healthcare is undeniable, the application of GPT-4 in this context is problematic for several reasons. Firstly, the authors seem to overlook the inherent complexity and specificity of operative notes. These notes are not merely generic descriptions of a surgical procedure; they are detailed accounts of a specific operation performed on a specific patient, taking into account the unique characteristics and circumstances of the case. A language model like GPT-4, no matter how advanced, cannot replicate the nuanced understanding and clinical judgment of a human surgeon.

Secondly, the authors fail to address the issue of accountability. Operative notes are legal documents that carry significant weight in the context of patient care and medico-legal disputes. The responsibility for the accuracy and completeness of these notes lies with the operating surgeon. Delegating this responsibility to an AI model raises serious ethical and legal questions.

Thirdly, the authors' assertion that GPT-4 can generate a "detailed note, with all the required components of a good ophthalmic note" is misleading. The example provided in the article is a generic description of a cataract surgery, lacking the specific details that would be expected in a real operative note. Furthermore, the authors do not provide any evidence of GPT-4's ability to accurately capture the unique aspects of individual cases, such as intraoperative complications or deviations from the standard procedure. Lastly, the authors' claim that GPT-4 can "drastically improve the time-consuming process of writing ophthalmic surgical notes" is unsubstantiated. While AI can certainly assist in automating certain tasks, the writing of operative notes requires a level of clinical understanding and decision-making that cannot be automated. The time saved by using GPT-4 to generate a generic operative note would likely be offset by the time required for the surgeon to review and correct the note.

Moreover, the application of AI in healthcare raises significant data protection issues that the authors fail to address. Operative notes contain sensitive patient information, and the use of AI models like GPT-4 in processing this data poses potential risks to patient privacy. The authors do not discuss how these risks would be mitigated, nor do they consider the implications of data protection regulations such as the General Data Protection Regulation (GDPR) in the EU. This oversight highlights the need for a more comprehensive exploration of the complexities involved in applying AI in healthcare.

In conclusion, while the application of AI in healthcare holds great promise, it is essential to approach this field with a clear understanding of the limitations of AI and the unique complexities of medical practice. The use of GPT-4 to generate surgical operative notes, as proposed by Waisberg et al., is in my view a misguided approach that overlooks these crucial considerations.

Sincerely,

Aaron Lawson McLean
